# Carbamazepine Toxicity Masquerading as Complex Febrile Seizures in a Pediatric Patient

**DOI:** 10.1155/2020/1790310

**Published:** 2020-03-16

**Authors:** Richard J Chen, Muhammad Ershad, Maricel Dela Cruz, Ahmed Mamdouh Taha Mostafa

**Affiliations:** Drexel University College of Medicine, Department of Emergency Medicine, Division of Medical Toxicology, Philadelphia, PA, USA

## Abstract

Carbamazepine is an antiepileptic drug that can cause seizures in overdose. In certain patient populations, this may be misdiagnosed as a seizure disorder. We describe a case of a 20-month-old female who presented with fever and seizure-like activity who was initially thought to have complex febrile seizures. Further historical information prompted carbamazepine level to be checked, which was found to be 29 mcg/ml (therapeutic range of 4–12 mcg/ml). Her carbamazepine levels downtrended with multidose activated charcoal. Her condition improved, and she was discharged without evidence of permanent neurologic sequelae. This case illustrates that xenobiotic exposure should often be considered, even if historical clues are not present, as they can often present as other conditions leading to misdiagnosis and delayed treatment.

## 1. Introduction

Carbamazepine (CBZ), known by the brand name Tegretol, is an iminostilbene compound. It is used in the management of seizure disorders, trigeminal neuralgia, and psychiatric illness, such as bipolar disorder and pain syndromes [1, 2]. Because of its pharmacologic properties, carbamazepine has the potential to be life threatening in overdose.

Carbamazepine functions by inhibition of sodium channels and interference with glutamate metabolism. As a result of this, CBZ has cardiotoxic properties and has significant effects on the CNS. In the pediatric population, where accidental ingestion and inability to verbalize events can cloud clinical diagnosis, it is especially dangerous because of its lower threshold of toxicity [3]. We present here a case of a 20-month-old presenting with status epilepticus due to carbamazepine toxicity.

## 2. Case

A 20-month-old previously healthy female was brought into the emergency department because of acute onset of altered mental status. The mother had noted that the patient also had episodes of flexion and extension of her upper extremities. The patient was then observed in emergency department to have tonic clonic seizure activity, which was responsive to lorazepam.

The patient was febrile at 104.5 F and tachycardic at 160 bpm and had a blood pressure of 111/76 and breathing 25 breaths/minute with an oxygen saturation of 100% on RA. The patient was noted to be lethargic, with pupils that were 2 mm in size, sluggishly reactive, bilaterally. Neurologically, the patient was observed to have decreased tone, midline gaze, and no facial asymmetry. She was not responsive to noxious stimuli. Deep tendon reflexes were noted to be 3+ bilaterally in the upper and lower extremities. Patient initial blood work was notable for hypokalemia (2.6 meq/L), metabolic acidosis, and elevated lactate of 6 mmol/L. The patient continued to have focal seizures not controlled by lorazepam.

The patient was subsequently admitted to the pediatric intensive care unit (PICU). An extensive workup was undertaken. The patient was empirically treated for severe infection. Her workup was notable for negative blood cultures, cerebral spinal fluid analysis, and negative computed tomography scan of the head and normal chest X-ray.

During her PICU stay, the patient's mother eventually revealed that her other daughter is prescribed carbamazepine and that a few pills were found to be missing. A CBZ level was sent by the primary team and noted to be elevated to 29 mcg/ml. Multidose activated charcoal (MDAC) was started.

Two days after MDAC was started, the patient's repeat CBZ levels were 20, 11, and <2 mcg/ml. The patient was extubated after, with no further episodes of seizure activity. The patient had no residual neurologic deficient and was eventually discharged home.

## 3. Discussion

This was a case of accidental poisoning with carbamazepine in a 20-month-old that resulted in status epilepticus that was initially misdiagnosed as complex febrile seizures. Unintentional/accidental nonfood poisoning in children <5 years old is best described by the term exploratory ingestion. This arises from the increased curiosity and sense of independence a child begins to gain as they grow older. There is usually an environmental component to increasing risk of ingestion, such as poor storage with easy access. Often, an underestimation of a child's physical ability plays a large part in poor storage practices [4].

Ingestion of >10 mg/kg of CBZ generally results in supratherapeutic levels. In overdose, carbamazepine primarily affects the central nervous system. At lower serum levels, patients will initially present with nystagmus, mydriasis, and tachycardia. At higher levels, patients can develop myoclonus and hyperthermia, become significantly more lethargic, and develop seizures, with progression to coma and respiratory arrest [5, 6]. Although the correlation between clinical symptoms and serum levels is poor, typical toxic serum concentrations are >20 mg/L, with cardiotoxicity more common at >40 mg/L, but in children, lower serum concentrations can result in serious toxicity [3, 5, 7].

Due to its structural similarity to tricyclic antidepressants, CBZ has significant cardiotoxic properties of sodium channel blockade and potassium channel blockade as well as anticholinergic effects. It can cause QRS widening and QTc prolongation as manifest on the EKG, predisposing to fatal dysrhythmias. Case reports note that QRS widening is often transient and may not result in clinical consequence [5, 7]. Cardiac monitoring is advised in patients presenting with severe toxicity.

Metabolism of CBZ mainly occurs by CYP3A4, creating the active metabolite carbamazepine-10,11-epoxide. This active metabolite has a longer half-life and is thought to contribute to toxicity. This fact potentially explains the lack of correlation between CBZ levels and clinical symptoms [8].

In chronic use, CBZ has been associated with bone marrow suppression, hepatitis, cardiomyopathy, renal disease, and increased risk for Stevens–Johnson syndrome [1, 5]. The adverse effects are more commonly reported in chronic use, with time of onset within 6 months of therapy [9]. In acute toxicity, patients present with central nervous system toxicity, cardiotoxicity, and anticholinergic effects.

Treatment of CBZ toxicity is mainly supportive. Airway and breathing should be addressed as required, including intubation if needed. Intravenous fluids and vasopressors should be given early in the setting of hypotension. Decontamination should be considered in the acute setting, and multidose activated charcoal is effective in preventing enterohepatic recirculation of CBZ. This can help reduce the elimination half-life, effectively speeding up the time it takes for a patient's burden of CBZ to decrease. Therefore, it is often recommended if there are no contraindications [5, 10].

## 4. Conclusions

In children, accidental ingestion of xenobiotics should always be considered. In pediatric patients who present with new onset status epilepticus, a detailed history including medications available at home should be obtained. In young children, even in the absence of a history suggesting xenobiotic exposure, accidental poisoning should remain on the differential. Failure to do so can lead to misdiagnosis and delayed treatment. Carbamazepine overdose can result in potentially life-threatening toxicity, secondary to cardiotoxicity and CNS toxicity, which may masquerade as complex febrile seizures, as in the patient presented here. Early identification prompts rapid intervention and treatment and will lead to improved patient outcomes.
